# Paxillin is an intrinsic negative regulator of platelet activation in mice

**DOI:** 10.1186/1477-9560-12-1

**Published:** 2014-01-02

**Authors:** Asuka Sakata, Tsukasa Ohmori, Satoshi Nishimura, Hidenori Suzuki, Seiji Madoiwa, Jun Mimuro, Kazuomi Kario, Yoichi Sakata

**Affiliations:** 1 Research Division of Cell and Molecular Medicine, Center for Molecular Medicine, Jichi Medical University School of Medicine, 3111-1 Yakushiji, Shimotsuke, Tochigi 329-0498, Japan; 2 Division of Cardiovascular Medicine, Department of Internal Medicine, Jichi Medical University School of Medicine, 3111-1 Yakushiji, Shimotsuke, Tochigi 329-0498, Japan; 3 Department of Cardiovascular Medicine, The University of Tokyo, 7-3-1 Hongo, Bunkyo, Tokyo 113-8655, Japan; 4 Translational Systems Biology and Medicine Initiative, The University of Tokyo, 7-3-1 Hongo, Bunkyo, Tokyo 113-8655, Japan; 5 Department of Morphological and Biomolecular Research, Nippon Medical School, 1-1-5 Sendagi, Bunkyo, Tokyo 113-8602, Japan

**Keywords:** Platelet, Glycoprotein, Platelet aggregation, Release reaction

## Abstract

**Background:**

Paxillin is a LIM domain protein localized at integrin-mediated focal adhesions. Although paxillin is thought to modulate the functions of integrins, little is known about the contribution of paxillin to signaling pathways in platelets. Here, we studied the role of paxillin in platelet activation in vitro and in vivo.

**Methods and results:**

We generated paxillin knockdown (Pxn-KD) platelets in mice by transplanting bone marrow cells transduced with a lentiviral vector carrying a short hairpin RNA sequence, and confirmed that paxillin expression was significantly reduced in platelets derived from the transduced cells. Pxn-KD platelets showed a slight increased in size and augmented integrin αIIbβ3 activation following stimulation of multiple receptors including glycoprotein VI and G protein-coupled receptors. Thromboxane A_2_ biosynthesis and the release of α-granules and dense granules in response to agonist stimulation were also enhanced in Pxn-KD platelets. However, Pxn-KD did not increase tyrosine phosphorylation or intracellular calcium mobilization. Intravital imaging confirmed that Pxn-KD enhanced thrombus formation in vivo.

**Conclusions:**

Our findings suggest that paxillin negatively regulates several common platelet signaling pathways, resulting in the activation of integrin αIIbβ3 and release reactions.

## Background

A breakdown of normal platelet function results in either unexpected bleeding or thrombotic events [1]. Platelets are inactive in the intact vasculature under physiological conditions. However, once the platelets encounter an injured region of the endothelium, they attach through an interaction between von Willebrand factor and the glycoprotein (GP) Ib/IX/V complex [2], and then collagen receptor GPVI triggers platelet activation. Activated platelets release several classes of agonists, including ADP and thromboxane (Tx) A_2_, which promote further platelet activation [3]. These steps ultimately increase the affinity of integrin αIIbβ3 for its ligands and induce platelet aggregation [4]. The intracellular signaling that increases the affinity of integrins is known as inside-out signaling [4]. Multiple signal transduction pathways from various receptors share common inside-out signaling cascades. For example, phosphoinositol hydrolysis, which leads to calcium mobilization and protein kinase C activation [5], and Rap1b activation are well-known signaling pathways that regulate integrin-mediated platelet functions [6].

To increase the affinity of integrin αIIbβ3, inside-out signaling pathways induce a drastic conformational change of the integrin [7]. Direct interactions between cytoskeletal proteins (e.g., talin and kindlin) and cytoplasmic β integrin are essential for inducing the conformational change of integrins [7]. Indeed, the loss of talin or kindlin in platelets dramatically reduces integrin αIIbβ3-mediated platelet aggregation, despite normal expression levels of the surface receptors [8,9]. Selective blockade of talin binding by a single amino acid substitution in β3 integrin also impairs integrin αIIbβ3-dependent platelet responses [10]. Although a number of integrin-associated proteins have been reported [11], the identities of proteins and their roles in regulating integrin signaling in platelets have not been fully characterized. It is also unknown whether additional molecules, other than talin and kindlin, are capable of regulating integrin signaling pathways.

Paxillin is a LIM domain protein that was originally identified as a substrate for oncogene *v-src*[12]. Paxillin contains two conserved structural domains, the N-terminus and C-terminus, which consist of four LIM domains [13,14]. Two other family members have also been identified, Hic-5 and leupaxin [13,14]. Paxillin is ubiquitously expressed alongside these variants [13,14], except in human platelets that predominantly express Hic-5 [15,16]. Conversely, mouse platelets express paxillin and leupaxin in addition to Hic-5 [17]. Considering the multiple interaction motifs located within its structure, paxillin appears to serve as a signaling platform for the recruitment of numerous regulatory proteins near integrins [13,14]. Paxillin directly interacts with the cytoplasmic domain of integrin α4 and α9, but not αIIb, and these interactions controls integrin-mediated cell migration and spreading [18,19].

Integrin αIIbβ3 in platelets is suitable for studies of integrin receptors because its ligand binding and signal transduction pathways are well characterized. Elucidating the intracellular proteins involved in the activation of integrin αIIbβ3 can provide a better understanding of the functions of integrins and might result in the discovery of new antithrombotic targets [20]. We previously reported that lentiviral vector-mediated short hairpin RNA (shRNA) expression in hematopoietic stem cells greatly reduces the expression of the target protein in platelets [21]. This method enables functional analyses of target proteins that modulate platelet activation in anucleate platelets [21]. In the present study, we used this method to investigate the roles of paxillin in platelet activation, and found that paxillin negatively regulates platelet signaling pathways including the activation of integrin αIIbβ3 and release reactions.

## Materials and methods

### Materials

All mouse cytokines were purchased from PeproTech (London, UK). The following antibodies and agonists were obtained from the specified suppliers: PAC-1 monoclonal antibody (mAb), anti-mouse P-selectin mAb (RB40.34), anti-paxillin mAb (clone 349), and anti-Hic-5 mAb (BD Biosciences, San Jose, CA); horseradish peroxidase-conjugated anti-green fluorescent protein (GFP) polyclonal antibody (Acris Antibodies, Himmelreich, Germany); phycoerythrin (PE)-Cy7-conjugated anti-mouse IgM (eBioscience, San Diego, CA); anti-talin mAb (clone 8D4); anti-phosphotyrosine mAb (clone 4G10), and BAPTA-AM (Millipore, Billerica MA); human fibrinogen and epinephrine (Sigma-Aldrich, St. Louis, MO); anti-vinculin mAb (V284) (Chemicon, Billerica, MA); anti-mouse GPVI mAb (Six.E10), anti-mouse GPIbα mAb (Xia.G5), and anti-mouse integrin αIIbβ3 mAb (Leo.D2 and clone JON/A) (Emfret Analytics, Eibelstadt, Germany); anti-α-actin mAb (D6F6), anti-FAK polyclonal antibody, and anti-Src mAb (32G6) (Cell Signaling Technology, Danvers, MA); anti-Rap1b polyclonal antibody and anti-protein kinase Cα mAb (M4) (Upstate Cell Signaling Solutions, Lake Placid, NY); allophycocyanin (APC)-conjugated anti-rat IgG polyclonal antibody (R& D Systems, Minneapolis, MN); convulxin (ALEXIS Biochemicals, Plymouth Meeting, PA); AYPGKF (Invitrogen, Carlsbad, CA); ADP (MC medical, Tokyo, Japan); U46619 (Cayman Chemical, Ann Arbor, MI).

### Lentiviral vector and virus production

A lentiviral vector plasmid for expression of shRNA sequences and GFP (LentiLox vector) was purchased from the American Type Culture Collection (Manassas, VA) [22]. To efficiently express GFP in platelets, the cytomegalovirus promoter of the LentiLox vectors was substituted with the platelet-specific GPIbα promoter (LentiLox-GPIbα) [21]. Putative shRNA sequences were designed using web-based software provided by Thermo Scientific Molecular Biology (http://www.thermoscientificbio.com/design-center/). Three shRNA sequences were synthesized for mouse paxillin and then cloned into a LentiLox vector plasmid (Additional files 1 and 2). Lentiviruses were produced as described previously [23].

### Transplantation of mouse bone marrow cells

**Figure 1:**
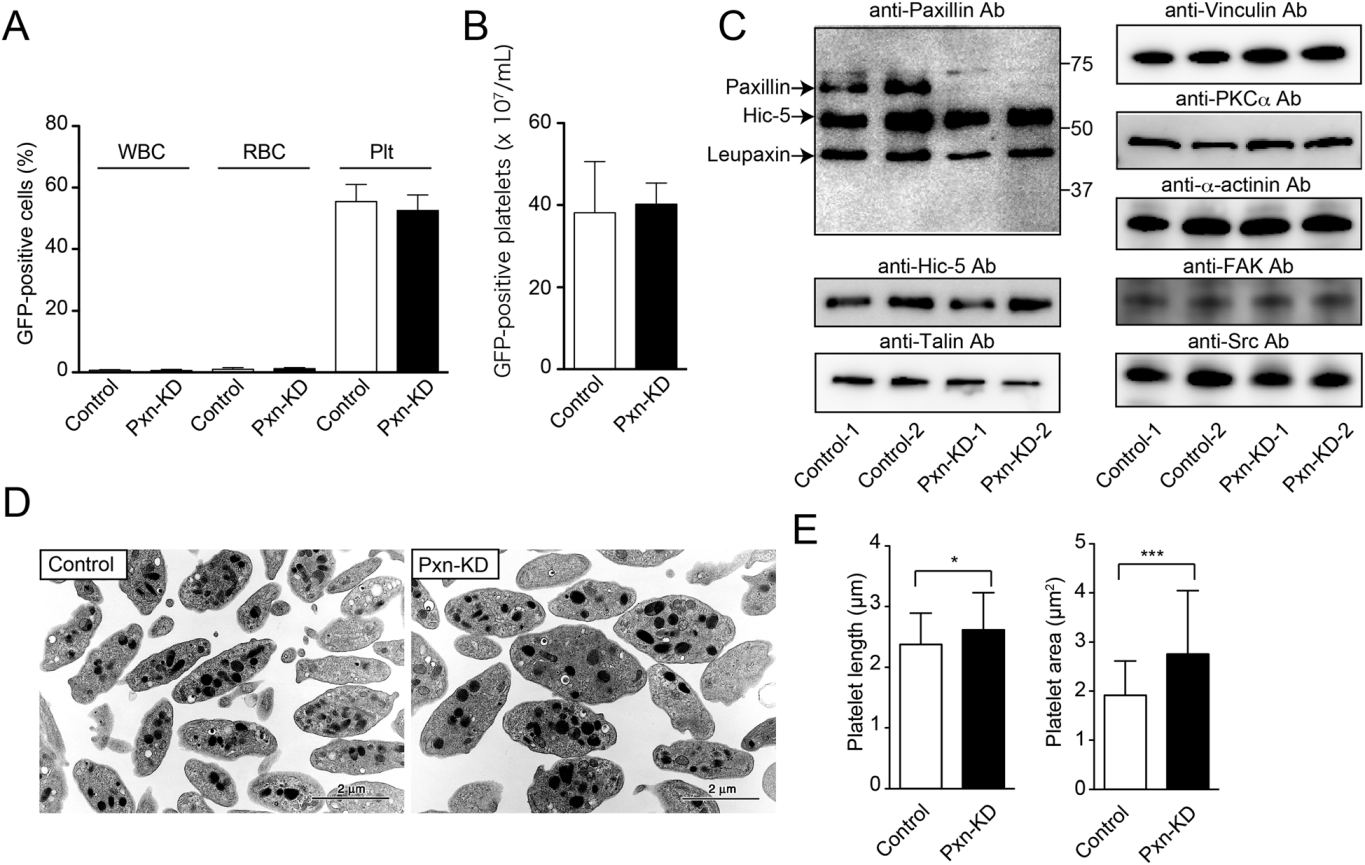
**Characterization of Pxn-KD platelets.** Bone marrow cells transduced with LentiLox-sh-control-GPIbα (Control) or LentiLox-sh-paxillin-GPIbα (Pxn-KD) at an MOI of 5 were transplanted into lethally irradiated recipient mice. **(A)** The numbers of GFP-positive cells (%) among white blood cells (WBCs), red blood cells (RBCs), and platelets (Plts) in peripheral blood at 30 days after transplantation were assessed by flow cytometry. Columns and error bars represent the mean ± s.d. (*n* = 10). **(B)** Absolute number of GFP-positive platelets in peripheral blood at 30 days after transplantation. Columns and error bars represent the mean ± s.d. (*n* = 10). **(C)** Platelet lysates were immunoblotted with the indicated antibodies. Data are for two mice with >80% of platelets expressing GFP in each experiment. **(D)** The morphology of control and Pxn-KD platelets was examined by transmission electron microscopy. Bars = 2 μm. **(E)** Platelet length (left panel) and area (right panel) were quantified by ImageJ software. Columns and error bars represent the mean ± s.d. (*n* = 53–72). Statistical significance was determined by the Student’s *t*-test. *P < 0.05 and ***P < 0.001 vs. control.

All animal procedures were approved by the Institutional Animal Care and Concern Committee of Jichi Medical University, and animal care was performed in accordance with the committee’s guidelines. Mouse bone marrow cells (C57BL/6 J) were isolated and resuspended in StemPro®-34 SFM medium (Invitrogen) supplemented with 100 ng/mL each of stem cell factor, thrombopoietin, interleukin-6, and fms-like tyrosine kinase 3 ligand, and 200 ng/mL soluble interleukin-6 receptor. The lentiviral vector was added at 12–16 h after cell isolation (multiplicity of infection [MOI] = 5), and the cell culture was continued for 21–22 h. Each recipient mouse (8–12 weeks of age) was irradiated with a single lethal dose of 9.5 Gy and then intravenously injected with 2 × 10^6^ lentivirus-transduced bone marrow cells. After transplantation, about 50% of platelets expressed GFP (Figure 1). Mice with 70% of their platelets exhibiting GFP positivity were used in experiments that could not distinguish GFP-positive platelets, *i.e.,* light transmission aggregometry, clot retraction, release concentration, calcium mobilization, and intravital microscopy.

### Immunoblotting

Immunoblotting with the specific antibodies was performed as described previously [21]. To assess protein tyrosine phosphorylation, washed platelets were pretreated with 1 mmol/L EDTA, 5 U/mL apyrase, and 10 μmol/L SQ29548 to exclude the effects of aggregation, released ADP, and TxA_2_.

### Transmission electron microscopy

Mouse platelet pellets were fixed in 2% glutaraldehyde in 0.1 mol/L phosphate buffer (pH 7.4) for 60 min at 4°C. The samples were washed, post-fixed with 1% osmium tetroxide in 0.1 mol/L phosphate buffer for 60 min at 4°C, dehydrated with a graded ethanol series, and then embedded in Epon (TAAB Laboratories, Aldermaston, UK) as described previously [24]. Ultrathin sections were prepared, stained with uranyl acetate and lead citrate, and then examined under a JEM1010 transmission electron microscope (JEOL, Tokyo, Japan) at an accelerating voltage of 80 kV. The length and area of platelets were quantified using ImageJ Ver. 10.2 for Macintosh (NIH, Bethesda, MD).

### Preparation of washed mouse platelets and flow cytometry

A blood sample (100–400 μL) was drawn from each mouse through the right jugular vein using a 30 G syringe containing 1/10 sodium citrate, and then diluted with 3 mL Hepes/Tyrode buffer (138 mmol/L NaCl, 3.3 mmol/L NaH_2_PO_4_, 2.9 mmol/L KCl, 1 mmol/L MgCl_2_, 1 mg/mL glucose, and 20 mmol/L Hepes, pH 7.4). The diluted blood was centrifuged at 120 × *g* for 8 min, and the platelets obtained from the platelet-rich fraction were washed and resuspended in Hepes/Tyrode buffer. Just prior to centrifugation, a 15% acid-citrate-dextrose A solution and 0.1 μmol/L prostaglandin I_2_ were added to inhibit platelet activation. The final platelet suspensions were adjusted to 1 × 10^7^ platelets/mL and supplemented with 1 mmol/L CaCl_2_. To assess the binding of JON/A, a monoclonal antibody (mAb) that recognizes activated mouse αIIbβ3 [25], to platelets, 30 μL of washed platelets was incubated with 4 μL of agonist solution, 4 μL of phycoerythrin (PE)-conjugated JON/A and 1 μL of biotin-conjugated anti-mouse P-selectin mAb for 5 min, and then supplemented with 1 μL of allophycocyanin (APC)-conjugated streptavidin. After 15 min of incubation, JON/A binding and P-selectin expression were determined by flow cytometry using a FACSAria Cell Sorter (Becton Dickinson, Mountain View, CA). Antibody binding was quantified as the mean fluorescence intensity (MFI) of GFP-positive platelets.

### Platelet aggregation

Washed platelets were prepared as described above. The final suspensions were adjusted to 2 × 10^8^ platelets/mL and supplemented with 1 mmol/L CaCl_2_ and 200 μg/mL fibrinogen. The aggregation response to agonist stimulation was measured based on light transmission measured using a PA-200 platelet aggregation analyzer (Kowa, Tokyo, Japan).

### Measurement of platelet products

Washed platelets (2 × 10^8^/mL) were stimulated with the indicated agonists for 15 min, and then the supernatants were recovered by centrifugation. The levels of platelet factor 4 (PF4) and serotonin in the supernatants were measured using a mouse PF4 enzyme-linked immunosorbent assay (ELISA) kit (R & D Systems) and an anti-serotonin ELISA kit (GenWay Biotech, San Diego, CA), respectively. The levels of TxB_2_ in the supernatants were measured using an enzyme immunoassay (Cayman Chemical).

### Platelet adhesion

Platelet adhesion to fibrinogen was assessed as described previously [21]. Briefly, eight-well dishes (Lab-Tek**®** Chamber Slide™) were coated with 400 μg/mL fibrinogen and then blocked with 1 mg/mL bovine serum albumin (BSA). Platelets were then added to the fibrinogen-coated dishes and incubated for 30 min at 37°C. Adherent platelets were fixed with 3% paraformaldehyde and then permeabilized with phosphate-buffered saline (PBS) containing 0.3% Triton X-100 and 5% donkey serum. After washing with PBS, the platelets were incubated with an anti-GFP polyclonal antibody (MBL, Aichi, Japan). Bound antibodies were detected by Alexa Fluor 488-conjugated anti-rabbit IgG. Actin filaments were detected by staining with 1 μg/mL rhodamine-conjugated phalloidin. Immunofluorescence staining was observed and photographed under a confocal microscope (FV1000; Olympus, Tokyo, Japan). The spread area of GFP-positive platelets was quantified using ImageJ software. Because Pxn-KD platelets were slightly larger than control platelets (Figure 1), the mean platelet size determined by BSA staining was subtracted from the total area on fibrinogen to calculate the actual increase in platelet spreading.

### Clot retraction

Human platelet-poor plasma was mixed with the same volume of Hepes/Tyrode buffer containing washed mouse platelets (final concentration: 3 × 10^8^ platelets/ml). Plasma coagulation was initiated by addition of 0.1 U/mL thrombin. The clots were photographed at various time points after thrombin addition. When indicated, 0.5 mmol/L manganese was added to exclude the role of inside-out signaling. The two-dimensional area of serum formation extruded by clot retraction was quantified using ImageJ software and expressed as the progression of clot retraction.

### Calcium mobilization

Platelets were incubated with GFP-Certified™ FluoForte™ dye (Enzo Life Sciences, Farmingdale, NY). The fluorophore-loaded platelets (2 × 10^8^/mL) were resuspended in Hepes-Tyrode buffer containing 1 mmol/L EDTA, 5 U/mL apyrase, and 10 μmol/L SQ29548 to exclude the effects of aggregation, extracellular calcium, released ADP, and TxA_2_. After stimulation, the intracellular calcium concentration was determined by monitoring the fluorescence (excitation, 530 nm; emission, 570 nm) using a microplate spectrofluorometer (Gemini EM; Molecular Devices, Sunnyvale, CA).

### Intravital microscopy and thrombus formation

Intravital microscopy was performed to analyze thrombus formation in vivo as reported previously [26]. Briefly, Texas Red-dextran (100 mg/kg body weight [BW], molecular weight: 70 kDa; Invitrogen), Hoechst 33342 (10 mg/kg BW; Invitrogen), Dylight 488-conjugated anti-CD42b antibody (200 μg/kg BW; Emfret), and hematoporphyrin (5 mg/kg BW; Sigma) were injected into anesthetized mice to produce reactive oxygen species (ROS) following laser irradiation. Blood cell dynamics were visualized during laser excitation (wavelengths 405, 488, and 561 nm; 1.5 mW total power at 100× objective lens). After laser irradiation, sequential images of the mesentery were obtained using a resonance scanning confocal microscope (Nikon A1R; Nikon, Tokyo, Japan). The areas of thrombus (shown by anti-CD42b antibody signals) before and after laser irradiation were calculated using NIS-Elements AR 3.2 (Nikon). When indicated, thrombus formation in the femoral artery was triggered by topical application of a filter paper tip saturated with 10% FeCl_3_. After injection of Texas Red-dextran, Hoechst 33342, and Dylight 488-conjugated anti-CD42b antibody, thrombus formation was visualized and monitored by confocal microscopy using two photon microscopy (excitation wavelength 840 nm) by NikonA1R MP (Nikon).

### Bleeding time

The distal tail tip (5 mm) of an anesthetized mouse was clipped, and the tail was immediately immersed in PBS at 37°C. Tail bleeding times were defined as the time required for the bleeding to stop.

## Results

### Generation of paxillin knockdown (Pxn-KD) platelets

To address the function of paxillin in mouse platelets, we used a lentiviral vector carrying shRNA sequences and GFP [22]. We synthesized three shRNA sequences for mouse paxillin, and cloned them into a LentiLox vector plasmid (Additional files 1 and 2). We selected one sequence that significantly inhibited paxillin expression in embryonic fibroblasts after transduction (Pxn-1 sequence; Additional files 1 and 2). After transplantation of bone marrow cells transduced with either the control or Pxn-KD sequence, about 50% of the platelets expressed GFP, and the absolute numbers of GFP-positive platelets did not differ between experiments using control and Pxn-KD sequences (Figure 1A–B). Furthermore, there was no effect on the total number of platelets (control: 6.8 ± 1.72 × 10^8^/mL; Pxn-KD: 7.7 ± 0.65 × 10^8^/mL, *P =* 0.18). We compared the platelet aggregation response and release reaction in platelets from wild-type C57BL/6 J and control mice, and confirmed that platelet aggregation as well as the release reaction did not differ (data not shown). To confirm knockdown of paxillin in GFP-positive platelets, we selected mice in which more than 80% of platelets expressed GFP after transplantation. Immunoblotting of platelet lysates with an anti-paxillin mAb (clone 349) showed a marked reduction in paxillin expression following transplantation of bone marrow cells transduced with the Pxn-KD sequence (Figure 1C). This antibody also recognizes other members of the paxillin family, including Hic-5 and leupaxin [17]. However, Hic-5 and leupaxilin were not affected by expression of the Pxn-KD sequence (Figure 1C). Transmission electron microscopy of resting platelets revealed that the Pxn-KD platelets were slightly larger than control platelets (Figure 1D–E). This change was largely dependent on an increase of the cytoplasm volume, but not the granule volume (Additional file 3). Pxn-KD platelets showed marginally elevated expression levels of GPIb and integrin αIIbβ3, even though GPVI expression was normal (Additional file 4). These changes in Pxn-KD platelets were supposed to result from the increase in platelet size.

### Augmentation of integrin αIIbβ3 activation in Pxn-KD platelets

**Figure 2:**
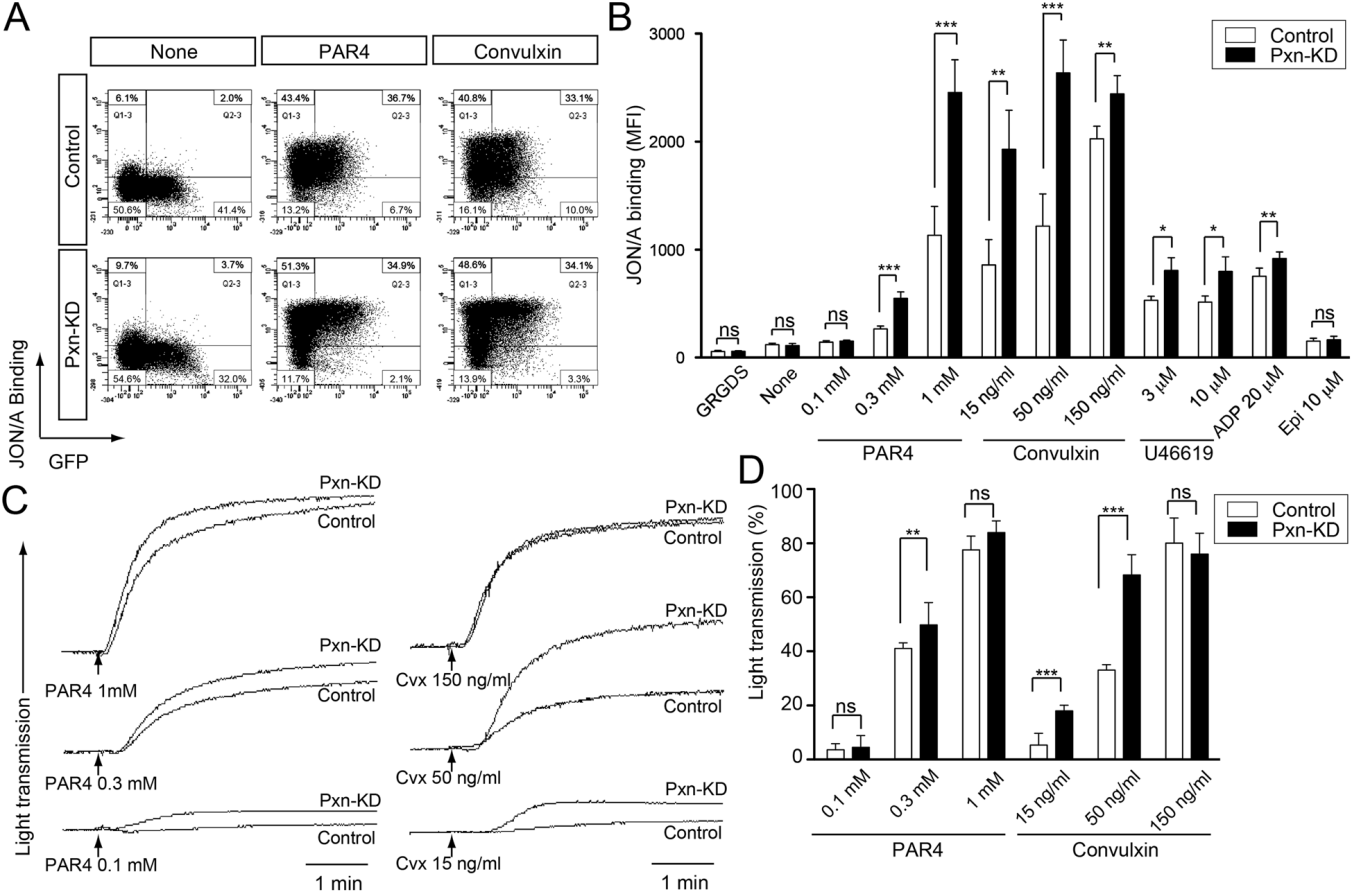
**Pxn-KD in platelets increases agonist-induced integrin αIIbβ3 activation.** Bone marrow cells transduced with LentiLox-sh-control-GPIbα (Control) or LentiLox-sh-paxillin-GPIbα (Pxn-KD) at an MOI of 5 were transplanted into lethally irradiated recipient mice. **(A)** Activation of integrin αIIbβ3 was assessed by JON/A binding to platelets incubated with or without 1 mmol/L AYPGKF or 50 ng/mL convulxin. The plots represent the degree of GFP expression (horizontal) and binding of JON/A, a monoclonal antibody that recognizes activated integrin αIIbβ3 (vertical). **(B)** Columns and error bars represent the mean ± s.d. of the MFI of JON/A binding after stimulating GFP-positive platelets with the indicated agonists (*n* = 3–5). **(C)** Platelet aggregation induced by the indicated concentration of AYPGKF or convulxin was monitored by light transmission aggregometry. **(D)** Columns and error bars represent the mean ± s.d. of maximal platelet aggregation after stimulation (*n* = 5). Open bars: control platelets; black bars: Pxn-KD platelets. Statistical significance was determined by the Student’s *t*-test. *P < 0.05, **P < 0.01, and ***P < 0.001.

We first focused on the role of paxillin in integrin αIIbβ3 activation that is critical for platelet aggregation. We performed flow cytometric analysis of integrin αIIbβ3 activation using an anti-JON/A mAb [25]. GFP-positive Pxn-KD platelets (Figure 2A, lower panel) showed significantly enhanced αIIbβ3 activation following stimulation compared with that of control platelets (Figure 2A, upper panel). Enhanced JON/A binding of Pxn-KD platelets was observed following stimulation with the GPVI agonist convulxin and G protein-coupled receptor agonists including a protease-activated receptor 4 agonist (AYPGKF), ADP, and U46619 (Figure 2A–B). However, JON/A binding was not enhanced in unstimulated or epinephrine-stimulated platelets, suggesting that Pxn-KD alone does not induce activation of integrin αIIbβ3. We next used light transmission aggregometry to assess platelet aggregation in vitro. We found that platelet aggregation was significantly augmented in Pxn-KD platelets, and this effect was evident at low agonist concentrations that induce platelet aggregation (Figure 2C–D).

### Enhanced release reactions and Tx biosynthesis in Pxn-KD platelets

**Figure 3:**
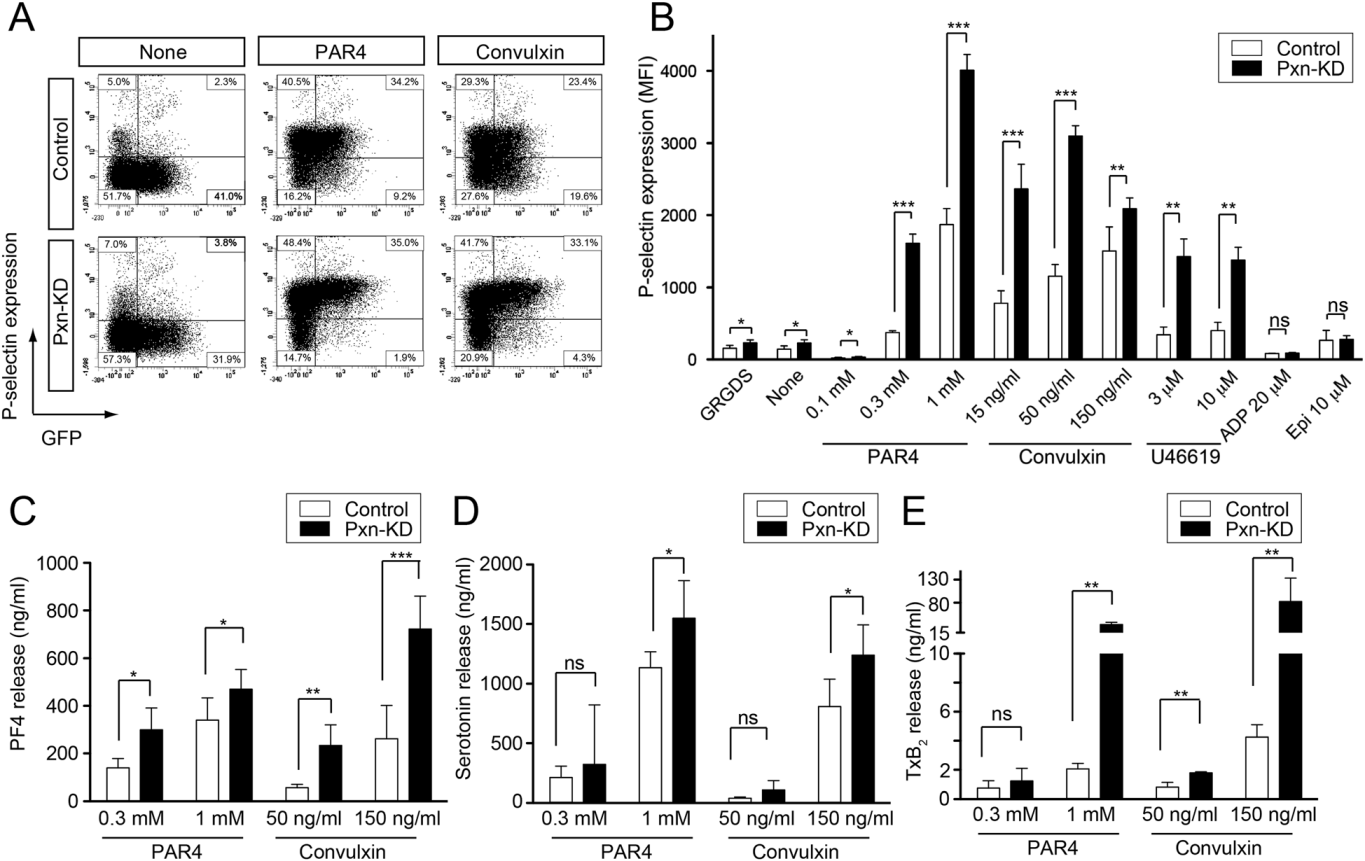
**The release reaction is enhanced in Pxn-KD platelets.** Bone marrow cells transduced with LentiLox-sh-control-GPIbα (Control) or LentiLox-sh-paxillin-GPIbα (Pxn-KD) at an MOI of 5 were transplanted into lethally irradiated recipient mice. **(A)** P-selectin expression on GFP-positive platelets stimulated with or without 1 mmol/L AYPGKF or 50 ng/mL convulxin was assessed by flow cytometry. The plots represent the degree of GFP expression (horizontal) and P-selectin expression (vertical). **(B)** Columns and error bars represent the mean ± s.d. of P-selectin expression after stimulation in GFP-positive platelets (*n* = 3–5). (C–E) Washed platelets were stimulated with the indicated agonist for 15 min, and then the concentrations of PF4 **(C)**, serotonin **(D)**, and TxB_2_**(E)** were measured in the supernatants. Columns and error bars represent the mean ± s.d. (*n* = 5). Open bars: control platelets; black bars: Pxn-KD platelets. Statistical significance was determined using Student’s *t*-test. *P < 0.05, **P < 0.01, and ***P < 0.001 vs. control.

We next assessed the release reactions in response to stimulation. To address the role of paxillin in α-granule secretion, P-selectin expression was determined in GFP-positive platelets by flow cytometry. As shown in Figure 3A–B, P-selectin expression in Pxn-KD platelets was significantly increased following stimulation with convulxin, AYPGKF, and U46619. In contrast, P-selectin expression was not increased by stimulation with ADP or epinephrine. We observed negligible increases in P-selectin expression of Pxn-KD platelets under the resting condition and after incubation with the fibronectin peptide Gly-Arg-Gly-Asp-Ser (GRGDS) (Figure 3B). To examine whether Pxn-KD platelets are already activated during circulation, we compared P-selectin expression in washed platelets and whole blood platelets before the preparation. An increase of P-selectin expression after washing the platelet preparation was observed in Pxn-KD platelets (30.0 ± 9.71 to 37.2 ± 5.72 in the control vs. 27.8 ± 2.56 to 44.8 ± 7.87, *P* < 0.05), suggesting that the susceptibility of Pxn-KD platelets caused marginal activation during washing. Although PF4 and serotonin content in resting platelets did not differ between control and Pxn-KD platelets (Additional file 3), the actual release of PF4 and serotonin into the supernatant in response to platelet activation was also enhanced in Pxn-KD platelets (Figure 3C–D). Of note, a marked increase in TxB_2_ biosynthesis was observed in Pxn-KD platelets (Figure 3E). Pretreatment with the ADP scavenger apyrase and thromboxane A_2_ receptor antagonist SQ29548 somewhat corrected the increase of JON/A binding in Pxn-KD platelets. This result suggests that the extent of the increase of integrin activation is partially dependent on the release reaction (Additional file 5). Collectively, these data suggest that paxillin negatively regulates platelet activation signaling pathways leading to integrin activation, release reactions, and Tx synthesis. It is possible that general pathway (s) involved in platelet activation were enhanced by Pxn-KD, because platelet activation was increased in response to several classes of activators including GPVI and G protein-coupled receptors.

### Assessment of outside-in signaling pathways in Pxn-KD platelets

**Figure 4:**
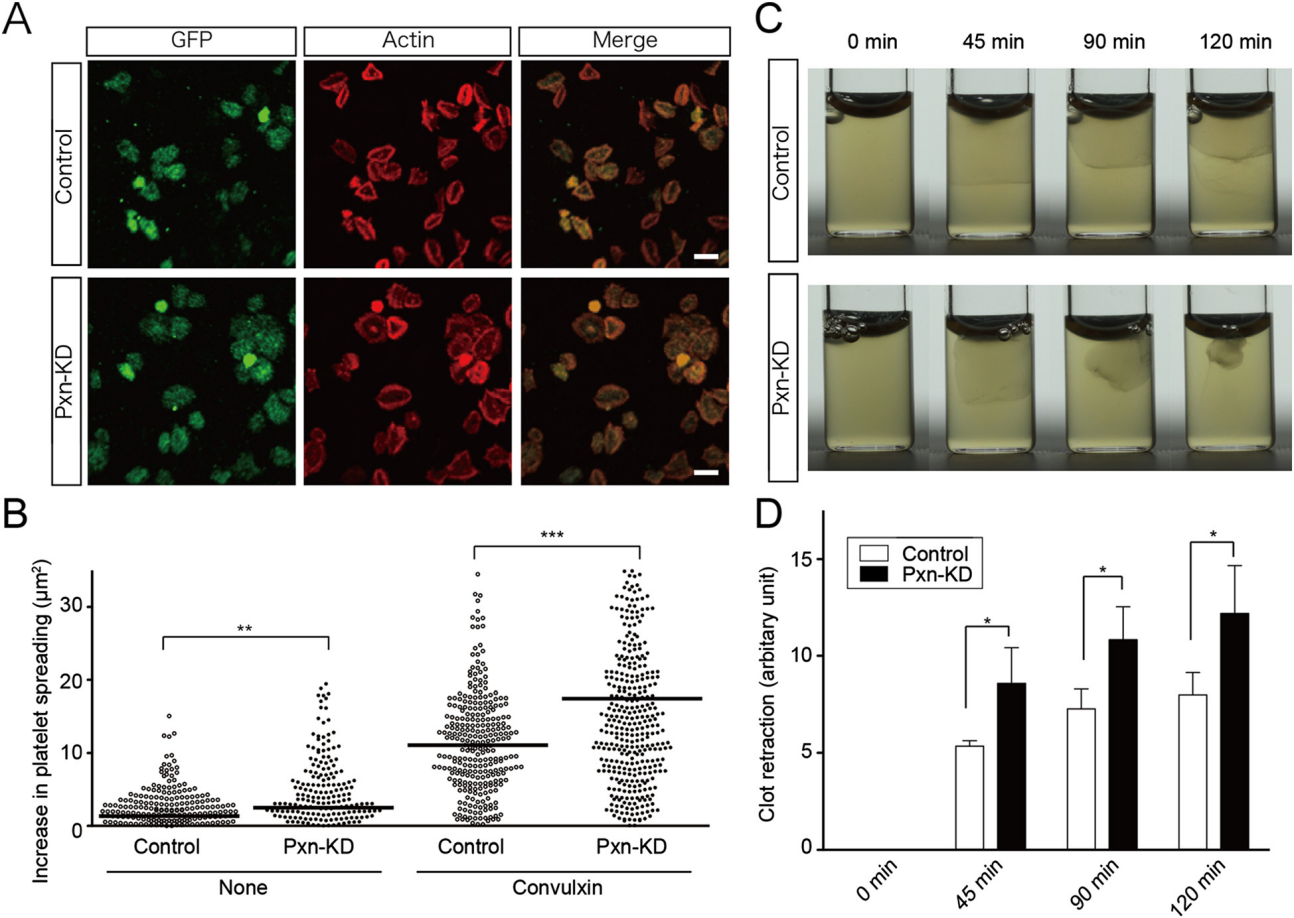
**Pxn-KD platelets exhibit augmented outside-in signaling. (A)** Control and Pxn-KD platelets treated with 50 ng/mL convulxin were allowed to adhere on immobilized fibrinogen for 30 min. The platelets were then fixed and stained with an anti-GFP antibody (green; left panel) and rhodamine-conjugated phalloidin (red; middle panel). The merged images show colocalization of GFP and actin staining (yellow; right panel). Original magnification, ×600; Bar, 5 μm. Data are representative of three independent experiments. **(B)** Platelets treated with or without 50 ng/mL convulxin were incubated in dishes coated with 400 μg/mL fibrinogen for 30 min. The area of cell spreading was quantified by ImageJ software. The mean platelet size on BSA was subtracted from the total spread area on fibrinogen to determine the actual increase in platelet spreading. The horizontal bar denotes the mean, and each symbol denotes an individual cell (*n* = 281–394 cells). **(C)** Clot retraction of platelet-rich plasma consisting of diluted human plasma and control or Pxn-KD platelets was initiated by 0.1 U/mL thrombin and then photographed at 0, 45, 90, and 120 min. **(D)** Clot retraction was quantified by measuring serum formation extruded by clot retraction. Columns and error bars represent the mean ± s.d. (*n* = 3). Open bars: control platelets; black bars: Pxn-KD platelets. Statistical significance was determined using Student’s *t*-test. *P < 0.05, **P < 0.01, and ***P < 0.001 vs. control.

To address the role of paxillin in outside-in signaling of integrin αIIbβ3, we assessed platelet spreading on fibrinogen and clot retraction. The cell area independent of integrin outside-in signaling (i.e., adherent to the BSA control) was slightly increased in Pxn-KD platelets compared with that in control platelets (data not shown), because the Pxn-KD platelets were marginally larger than control platelets (Figure 1). To quantify the increase in platelet spreading, the mean platelet size on BSA was subtracted from the total spreading area on fibrinogen. As shown in Figure 4A, the increase in platelet spreading on fibrinogen without or with convulxin stimulation was significantly greater for Pxn-KD platelets than that for control platelets (Figure 4A–B). In addition, clot retraction induced by thrombin was significantly enhanced in Pxn-KD platelets compared with that in control platelets (Figure 4C–D). Acceleration of clot retraction in Pxn-KD platelets was also observed in the presence of manganese at 15 min (6.98 ± 0.130 vs. 7.56 ± 0.072, *P* < 0.05). These observations suggest that paxillin is an important regulator of integrin outside-in signaling via integrin αIIbβ3.

### The role of paxillin in calcium mobilization in platelets

**Figure 5:**
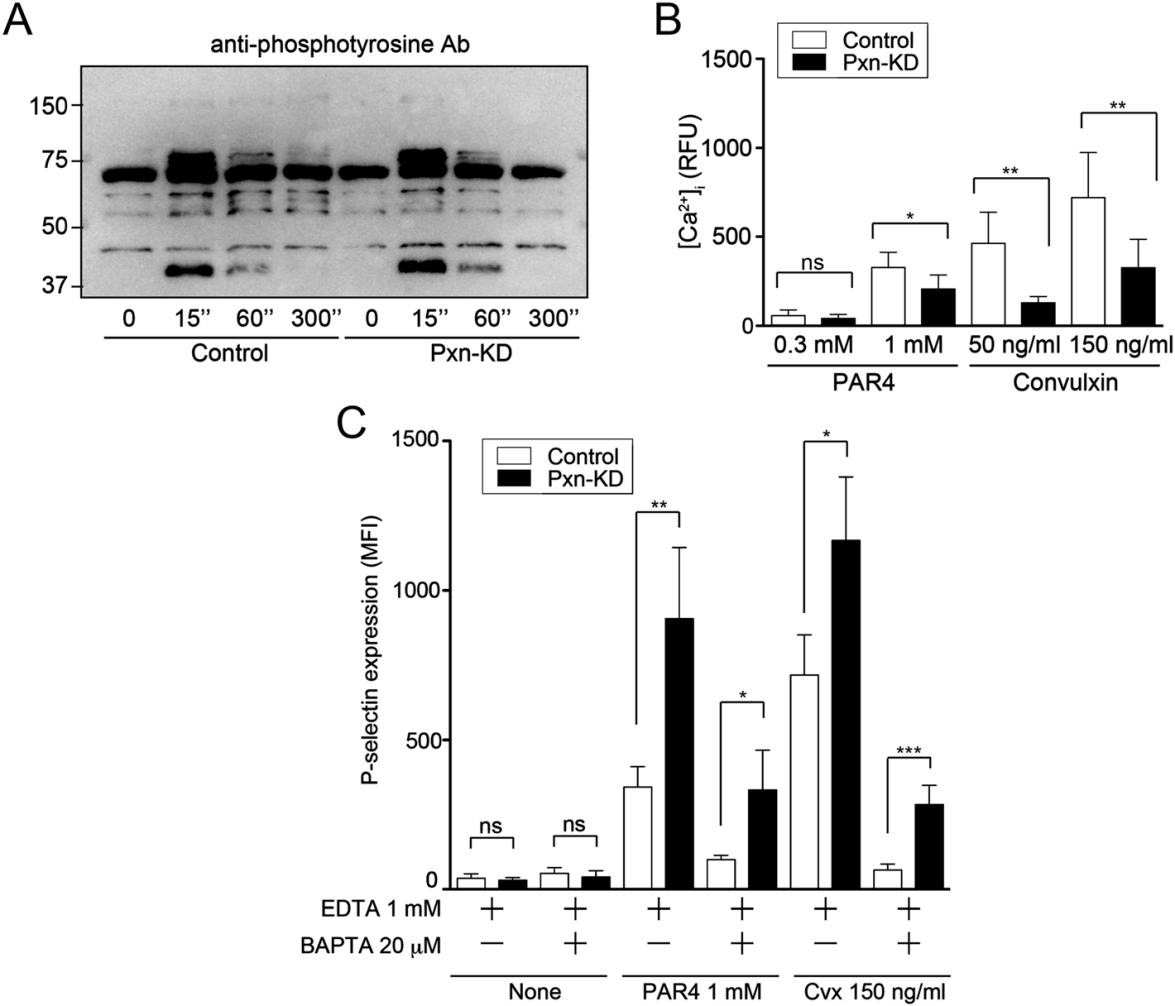
**Pxn-KD fails to increase tyrosine phosphorylation and calcium mobilization. (A)** Washed platelets obtained from control and Pxn-KD experiments were stimulated with 150 ng/mL convulxin for the indicated times. The cell lysates were resolved by SDS-polyacrylamide gel electrophoresis and then immunoblotted with an anti-phosphotyrosine mAb (4G10). The data shown are representative of three independent experiments. **(B)** Control and Pxn-KD platelets were labeled with GFP-Certified™ FluoForte™ dye. Changes in intracellular calcium levels after stimulation with an indicated concentration of AYPGKF or convulxin were then measured every 30 s. Data are expressed as the relative fluorescence unit (RFU) measured using a microplate spectrofluorometer (excitation, 530 nm; emission, 570 nm). The peak calcium concentration was measured after stimulation (open bars: control platelets; black bars: Pxn-KD platelets). Columns and error bars represent the mean ± s.d. (*n* = 5–8). Statistical significance was determined by the Student’s *t*-test. **(C)** Control and Pxn-KD platelets were pretreated with 1 mmol/L EDTA and/or 20 μmol/L BAPTA-AM for 10 min, and then stimulated with or without 1 mmol/L AYPGKF or 150 ng/mL convulxin. P-selectin expression on GFP-positive platelets was determined by flow cytometry. Columns and error bars represent the mean ± s.d. of P-selectin expression (n = 4).

Because GPVI initiates signaling cascades by activation of non-receptor tyrosine kinases, we assessed tyrosine phosphorylation elicited by the GPVI signaling pathway. As a result, tyrosine phosphorylation events induced by convulxin were not affected by Pxn-KD (Figure 5A). The agonist-induced increase in intracellular calcium mobilization is an important common and proximal signaling event controlling platelet activation. Therefore, we next examined whether Pxn-KD enhanced intracellular calcium mobilization following stimulation. To exclude secondary effects of platelet aggregation, influx of extracellular calcium, and release reactions, we preincubated the platelets with EDTA, apyrase, and SQ29548. Intracellular calcium mobilization induced by the GPVI agonist convulxin and G protein-coupled receptor stimulation with AYPGKF was rather decreased by Pxn-KD (Figure 5B). These data suggest that paxillin targets downstream signaling of calcium mobilization or a calcium-independent signaling pathway.

To explore the importance of calcium-independent signaling pathways in Pxn-KD platelets, we employed BAPTA-AM, an intracellular calcium chelator, to exclude the effect of calcium mobilization. Because JON/A requires extracellular calcium for antibody binding, we assessed P-selectin expression induced by an agonist. Pretreatment with BAPTA-AM significantly suppressed P-selectin expression in both control and Pxn-KD platelets (Figure 5C). On the other hand, P-selectin expression elicited by an agonist was still observed in Pxn-KD platelets even in the presence of BAPTA-AM (Figure 5C). These data indicate that downstream signaling from intracellular calcium mobilization is amplified by Pxn-KD, and the calcium-independent pathway is activated by Pxn-KD to increase platelet activation.

### Pxn-KD augments platelet adhesion and thrombus formation in vivo

**Figure 6:**
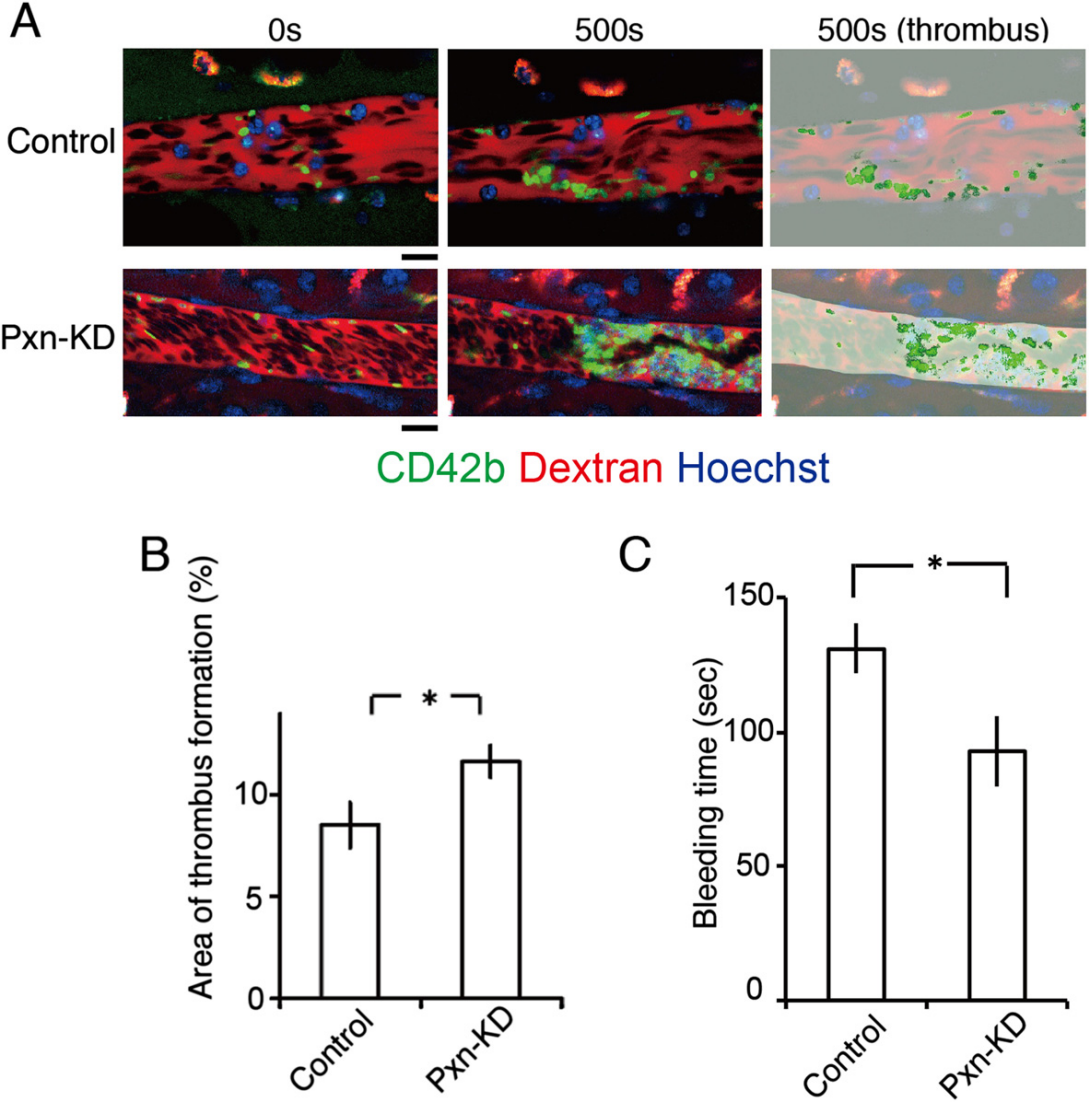
**Pxn-KD in platelets expedites thrombus formation in vivo. (A)** Intravital imaging of thrombus formation by laser irradiation of mesenteric arterioles in mice with control or Pxn-KD platelets. Thrombus formation was increased in mice with Pxn-KD platelets following laser irradiation. Bar, 10 μm. The right panel shows the results of quantification of the thrombus area. **(B)** Percentage areas of thrombus within blood vessels after laser irradiation. Columns and error bars represent the mean ± s.e.m. (*n* = 40 vessels in five mice/group). **(C)** Tail bleeding times were assessed as described in the Methods. Columns and error bars represent the mean ± s.e.m. (n = four mice/group). Statistical significance was determined using Student’s *t*-test. *P < 0.05 vs. control.

Finally, we examined the contribution of paxillin to thrombus formation in vivo. To visualize thrombus formation in vivo, we used a direct visual technique based on confocal microscopy in mesenteric capillaries [26]. Thrombus formation in this system was initiated by the production of ROS following laser irradiation [26]. Laser irradiation-induced thrombus formation was significantly enhanced in Pxn-KD platelets (Figure 6A and 6B and Additional files 6 and 7). In addition, there was an enhancement of thrombus formation initiated by FeCl_3_ in large femoral arteries (Additional file 8). Moreover, bleeding times after tail clipping significantly shortened in Pxn-KD experiments (Figure 6C). These findings support our hypothesis that paxillin is an important negative regulator of platelet activation and thrombus formation in vivo.

## Discussion

Here, we found that the LIM protein paxillin is a negative regulator of platelet activation in mice. The negative regulation of platelet activation by paxillin was not limited to a specific signaling pathway, because Pxn-KD enhanced platelet activation in response to a variety of agonists. We also confirmed that thrombus formation was augmented in Pxn-KD platelets in vivo. This finding is notable because several previous reports suggest that changes in paxillin function actually reduce integrin signaling [13,14]. Furthermore, a previous finding in platelets has demonstrated the possible role of paxillin as a negative feedback regulator after integrin ligation to regulate the activity of Lyn tyrosine kinase [17]. However, this mode of regulation cannot fully explain the phenotypes of Pxn-KD platelets, because both outside-in and inside-out signaling were augmented by Pxn-KD. Our results reveal a new cellular function of paxillin and indicate new mechanisms that modulate platelet activation.

The most interesting result of this study was that Pxn-KD significantly enhanced the upstream signaling pathways that converge on platelet activation. Appropriate inhibition of the platelet response is essential to control pathological thrombus formation. It is well known that the mediators that enhance intracellular cAMP or cGMP levels, including prostacyclin, prostaglandin E_1_, and nitric oxide, are strong extrinsic inhibitors of platelet activation [27]. These extrinsic mediators ameliorate the broad platelet activation elicited by various agonists [27]. Intrinsic negative regulators of platelet activation have been identified recently, but many of these proteins only control a specific receptor signaling pathway. GPVI-mediated immunoreceptor tyrosine-based activation motif (ITAM) signaling is regulated by immunotyrosine-based inhibitory motif (ITIM)-containing receptors including platelet endothelial cell adhesion molecule 1 and carcinoembryonic antigen-related cell adhesion molecule 1 [28,29]. Furthermore, Lyn tyrosine kinase has been reported to inhibit ITAM signaling by inducing tyrosine phosphorylation of ITIM [28]. It has also been reported that binding of a regulator of G-protein signaling to the Giα subunit limits platelet responsiveness to the receptor, which is independent of Rap1b [30]. Conversely, paxillin may downregulate platelet activity by modulating a common pathway, because Pxn-KD resulted in marked platelet hyperactivation in response to stimulation of tyrosine phosphorylation-based receptors and G protein-coupled receptors.

Although paxillin is reportedly involved in various integrin-mediated cellular functions, many of these functions are limited to outside-in signaling pathways. Paxillin-deficient embryos show embryonic lethality, and the phenotype closely resembles that of fibronectin-deficient mice [31]. Moreover, paxillin-deficient fibroblasts show reductions in cell migration and tyrosine phosphorylation following cell adhesion [31]. Chimeric integrin αIIbβ3 with a cytoplasmic tail substitution of α4β1 or α9β1, which facilitates paxillin binding, significantly inhibits cell spreading, but does not affect αIIbβ3-dependent cell adhesion [18,19]. Inhibition of paxillin binding to integrin α4 inhibits leukocyte recruitment to an inflammatory site [32]. These data suggest important roles of paxillin in outside-in signaling by direct interaction with the integrin α-subunit. However, in this study, inside-out and outside-in signaling of integrin αIIbβ3 were increased in Pxn-KD platelets, even though paxillin failed to interact with platelet-specific integrin αIIb [19]. It is possible that other signaling pathways in platelets are modulated by paxillin, which is independent of direct interactions with integrins.

An issue that remains unresolved is the precise mechanism governing the negative regulatory function of paxillin in platelet activation. As described above, Rathore et al. previously reported that integrin αIIbβ3-dependent platelet aggregation induced tyrosine phosphorylation of paxillin and Hic-5 in platelets, leading to the binding of Csk, which controls activation of the Src family of tyrosine kinases [17]. Csk preferentially binds to paxillin in murine platelets that coexpress paxillin and Hic-5 [17]. Furthermore, the interaction abolishes the activity of Lyn, but not Fyn or Src. It is possible that paxillin acts as a negative feedback regulator of outside-in signaling by modulating Lyn activity after ligand binding to integrin αIIbβ3 [17]. However, this mechanism does not fully explain the functional roles of paxillin in platelets. Our data suggest that paxillin controls additional proximal signaling pathways for platelet activation. Pxn-KD did not directly augment the conformational changes of integrin αIIbβ3 expressed on Chinese hamster ovary cells (Additional file 9), tyrosine phosphorylation, or calcium mobilization induced by phosphoinositide turnover. These data suggest that paxillin negatively controls downstream signaling of calcium mobilization or a calcium-independent signaling pathway. In addition, calcium mobilization was rather reduced by Pxn-KD. It is therefore possible that negative feedback exists to prevent further activation of Pxn-KD platelets, or phosphoinositide turnover is directly modulated by Pxn-KD.

Our data suggest that several mechanisms may increase platelet activation by Pxn-KD. Notably, calcium-independent actions by Pxn-KD appear to exist, because P-selectin expression elicited by an agonist was still observed in Pxn-KD platelets even in the presence of BAPTA-AM. A previous report has suggested that coordinated signaling through both G_12/13_ and G_i_ causes integrin αIIbβ3 activation, despite a small increase in intracellular calcium [33]. In addition, G_12/13_ and G_i_ signaling activates integrin αIIbβ3 in Gq-deficient mice [34]. It is possible that paxillin modify the calcium-independent signaling pathway leading to release reaction and integrinαIIbβ3 activation. Additional studies are needed to investigate how paxillin regulates platelet activation, and to assess whether these roles of paxillin in control of cellular signaling are common mechanisms in other cell types. We are now interested in further investigation of the precise mechanisms, and additional experiments are currently underway in our laboratory.

Another interesting finding of our study is that Pxn-KD resulted in an enlargement of platelet volume. CLP36, a member of the LIM domain family, was recently reported to play some roles in platelet activation [35]. Platelets from mice lacking the LIM domain of CLP36 show a slight increase in size and hyperactivation in response to a GPVI agonist [35]. The phenotypes of CLP36-deficient or mutant platelets are similar to those of Pxn-KD platelets in our study, although G protein-coupled receptor signaling is not affected in CLP36-deficient or mutant mice. Accordingly, the expression of LIM domain proteins may determine platelet size and reactivity.

To extrapolate the implications of our study to the biology and pathophysiology of humans, we must consider the differential expression pattern of paxillin-related proteins in platelets among species. Murine platelets express paxillin, Hic-5, and leupaxin, whereas human platelets only express Hic-5 [17]. Hagmann et al*.* reported that a switch from paxillin to Hic-5 expression should occur during the late phase of megakaryopoiesis in humans [15]. A recent report has described platelet functions in Hic-5-deficient mice [36]. Hic-5-deficient mice exhibit prolonged bleeding times, and the loss of Hic-5 in platelets slightly impairs integrin αIIbβ3 activation induced by thrombin, but not other agonists including convulxin, U46619, and ADP [36]. Although the hemostatic defect in Hic-5-deficient mice, as assessed by tail bleeding, is not fully explained by a mild defect in platelet function, it is possible that the structurally related proteins paxillin and Hic-5 play opposing roles in the regulation of platelet function in murine platelets. Leupaxin, another LIM protein that is predominantly expressed in leukocytes, has been reported to play an inhibitory role in B cell receptor signaling [37], which is similar to the role of paxillin reported in this study. In human platelets, which only express Hic-5, it will be necessary to elucidate whether Hic-5 acts as a positive regulator of integrin αIIbβ3 activation.

In summary, we have shown that paxillin is a negative regulator of platelet activation in mouse platelets. Modulation of platelet activation by Pxn-KD may originate in the augmentation of common signaling pathways, leading to integrin αIIbβ3 activation, release reactions, and Tx biosynthesis. Modulation of the LIM protein function might be an attractive candidate therapeutic target capable of strongly suppressing unexpected platelet activation in thrombotic disorders. The next challenge will be elucidating the precise mechanism by which paxillin regulates the signaling pathway in platelet activation.

## Abbreviations

Pxn-KD: Paxillin-knockdown; Tx: Thromboxane; shRNA: Short hairpin RNA; GRGDS: Gly-Arg-Gly-Asp-Ser; ROS: Reactive oxygen species; ITAM: Immunoreceptor tyrosine-based activation motif; ITIM: Immunotyrosine-based inhibitory motif.

## Competing interests

The authors declare that they have no competing interests.

## Authors’ contributions

Contribution: AS, TO, and SN designed the study, performed the experiments, analyzed the data, and wrote the manuscript; HS performed the experiments and wrote the manuscript; SM, JM, KK, and YS analyzed the data and revised the manuscript. All authors read and approved the final manuscript.

## Supplementary Material

Additional file 1 **Schematic diagrams of the lentiviral vector used in this study.** (A) Schematic diagram of the lentiviral vector. (B) Locations of the oligonucleotides encoding the shRNAs in the mouse *paxillin* (*Pxn*) gene. (C) Mouse embryonic fibroblasts were transduced with a lentiviral vector containing the control, Pxn-1, Pxn-2, or Pxn-3 shRNA sequences at MOIs of 1, 3, or 10. Protein expression was determined by immunoblotting at 48 h after transduction. Data are representative of three independent experiments. Click here for file

Additional file 2 Oligonucleotide sequences of siRNA cloned into LentiLox. Click here for file

Additional file 3 **Pxn-KD does not affect granule contents.** Bone marrow cells transduced with LentiLox-sh-control-GPIbα (Control) or LentiLox-sh-paxillin-GPIbα (Pxn-KD) at an MOI of 5 were transplanted into lethally irradiated recipient mice. (A) The morphology of control and Pxn-KD platelets was examined by transmission electron microscopy, and the areas of granules and cytoplasm in each platelet were independently quantified using ImageJ software for Macintosh. Columns and error bars represent the mean ± s.d. (*n* = 53–70). (B–C) Washed platelets were lysed to measure the concentrations of platelet factor 4 (PF4) (B) and serotonin (C). Columns and error bars represent the mean ± s.d. (*n* = 4). Statistical significance was determined using Student’s *t* test. ***P < 0.001 vs. control. Click here for file

Additional file 4 **Expression levels of platelet-specific glycoproteins.** Description of data: (A) Expression levels of GPIIb/IIIa (integrin αIIbβ3) (left panel), GPIb (middle panel), and GPVI (right panel) in control (dark gray) and paxillin-knockdown platelets (light gray). (B) Columns and error bars represent the mean ± s.d. of the mean fluorescence intensity (MFI) of antibody binding (*n* = 5). Statistical significance was determined using Student’s *t* test. *P < 0.05, **P < 0.01, and ***P < 0.001 vs. control. Click here for file

Additional file 5 **Effects of apyrase and SQ29548 on agonist-induced integrin αIIbβ3 activation and P-selectin expression in control and Pxn-KD platelets.** Platelets pretreated without or with 5 U/mL apyrase and 10 μmol/L SQ29548 were stimulated with the indicated agonists. JON/A binding (A) and P-selectin expression (B) on GFP-positive platelets were assessed by flow cytometry. Column and error bars represent the mean ± s.d. of the mean fluorescence intensity (MFI) (*n* =3–4). Statistical significance was determined using Student’s *t* test. *P < 0.05, **P <0.01, and ***P < 0.001 vs. control. Click here for file

Additional file 6 Intravital imaging of thrombus formation by laser irradiation of mesenteric arterioles in mouse with control platelets. Click here for file

Additional file 7 Intravital imaging of thrombus formation by laser irradiation of mesenteric arterioles in mice with Pxn-KD platelets. Click here for file

Additional file 8 **Thrombus formation in femoral arteries induced by FeCl3.** (A) Intravital imaging of thrombus formation 5 mins after FeCl3 treatment in femoral arteries in mice with control or paxillin knock-down platelets (Pxn-KD). The black arrows indicate the direction of blood flow, and triangles show the developed thrombus. Bar, 100 μm. (B) Areas of thrombus within arteries 20 mins after laser irradiation. Columns and error bars represent the mean ± s.e.m. (n = 8 arteries in four mice/group). Click here for file

Additional file 9 **Knock-down of paxillin does not affect talin-dependent activation of integrin αIIbβ3 in CHO cells.** (A) Schematic representation of the lentiviral vectors used in this experiment. (B–D) αIIbβ3-CHO cells were transduced with lentiviral vectors expressing a control shRNA sequence and GFP (Control), the paxillin shRNA sequence and GFP (Pxn-KD), a control shRNA sequence and the GFP-Talin FERM domain (Control-FERM), or the paxillin shRNA sequence and the GFP-Talin FERM domain (Pxn-KD-FERM). (B) Lysates obtained from the transduced cells were immunoblotted with anti-GFP polyclonal antibody, anti-paxillin monoclonal antibody, and anti-vinculin monoclonal antibody. (C) PAC-1 binding after transduction in the presence or absence of 1 mmol/L GRGDS was assessed by flow cytometry. Data are representative of four independent experiments. (D) Columns and error bars represent the mean ± s.d. of PAC-1 binding (n = 4). Statistical significance was determined using Student’s *t* test. Click here for file
