# Supplementary material for: Paxillin is an intrinsic negative regulator of platelet activation in mice
Source: Thromb J. 2014 Jan 2;12:1. doi: 10.1186/1477-9560-12-1 (PMC3904695; doi:10.1186/1477-9560-12-1)
Supplement: Additional file 1 — Schematic diagrams of the lentiviral vector used in this study. (A) Schematic diagram of the lentiviral vector. (B) Locations of the oligonucleotides encoding the shRNAs in the mouse paxillin (Pxn) gene. (C) Mouse embryonic fibroblasts were transduced with a lentiviral vector containing the control, Pxn-1, Pxn-2, or Pxn-3 shRNA sequences at MOIs of 1, 3, or 10. Protein expression was determined by immunoblotting at 48 h after transduction. Data are representative of three independent experiments. [file 1477-9560-12-1-S1.pdf]

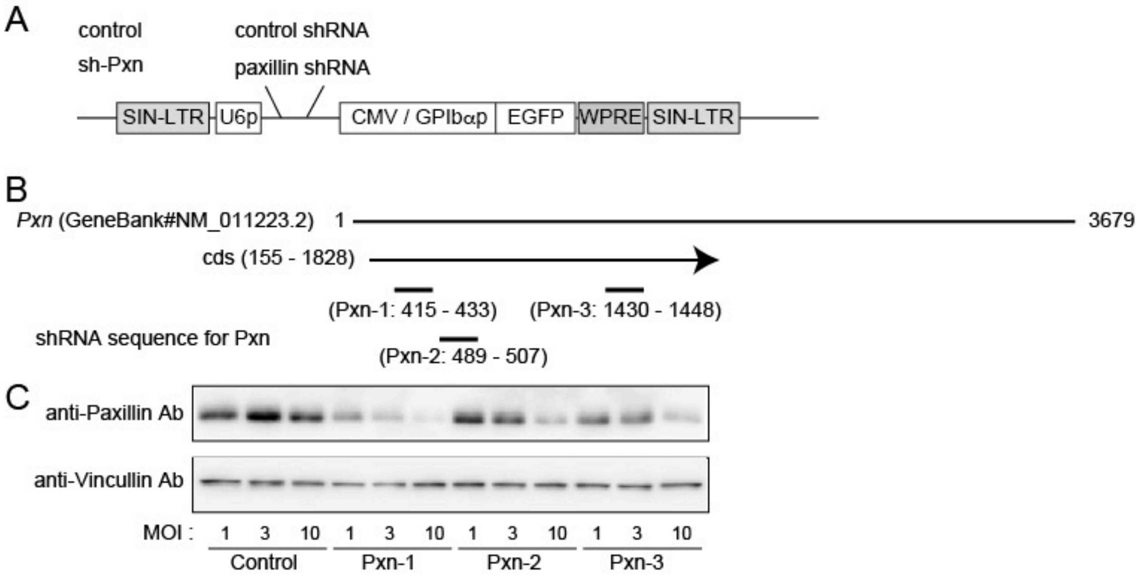

Additional File 1. Schematic diagrams of the lentiviral vector used in this study. (A) Schematic diagram of the lentiviral vector. (B) Locations of the oligonucleotides encoding the shRNAs in the mouse *paxillin* (*Pxn*) gene. (C) Mouse embryonic fibroblasts were transduced with a lentiviral vector containing the control, Pxn-1, Pxn-2, or Pxn-3 shRNA sequences at MOIs of 1, 3, or 10. Protein expression was determined by immunoblotting at 48 h after transduction. Data are representative of three independent experiments.
