# Supplementary material for: Paxillin is an intrinsic negative regulator of platelet activation in mice
Source: Thromb J. 2014 Jan 2;12:1. doi: 10.1186/1477-9560-12-1 (PMC3904695; doi:10.1186/1477-9560-12-1)
Supplement: Additional file 2 — Oligonucleotide sequences of siRNA cloned into LentiLox. [file 1477-9560-12-1-S2.pdf]

Additional Table 1. Oligonucleotide sequences of siRNA cloned into LentiLox

siRNA		Sequence
control	sence	5’- TGCTCGAATAGTACTAGAGTTTCAAGAGAGCTCTAGTACTATTCGAGCTTTTTTC -3’
	antisence	5’- TCGAGAAAAAAGCTCGAATAGTACTAGAGCTCTCTTGAAACTCTAGTACTATTCGAGCA -3’
Pxn-1	sence	5’- TGTACAGCTCCAGTGCTAAATTCAAGAGATTTAGCACTGGAGCTGTACTTTTTTC –3’
	antisence	5’- TCGAGAAAAAAGTACAGCTCCAGTGCTAAATCTCTTGAATTTAGCACTGGAGCTGTACA -3’
Pxn-2	sence	5’- TGCGAGGAAGAGCACGTCTATTCAAGAGATAGACGTGCTCTTCCTCGCTTTTTTC -3’
	antisence	5’- TCGAGAAAAAAGCGAGGAAGAGCACGTCTATCTCTTGAATAGACGTGCTCTTCCTCGCA -3’
Pxn-3	sence	5’- TGGCAAAGCGTACTGTCGTATTCAAGAGATACGACAGTACGCTTTGCCTTTTTTC -3’
	antisence	5’- TCGAGAAAAAAGGCAAAGCGTACTGTCGTATCTCTTGAATACGACAGTACGCTTTGCCA -3’
