# Supplementary material for: Paxillin is an intrinsic negative regulator of platelet activation in mice
Source: Thromb J. 2014 Jan 2;12:1. doi: 10.1186/1477-9560-12-1 (PMC3904695; doi:10.1186/1477-9560-12-1)
Supplement: Additional file 3 — Pxn-KD does not affect granule contents. Bone marrow cells transduced with LentiLox-sh-control-GPIbα (Control) or LentiLox-sh-paxillin-GPIbα (Pxn-KD) at an MOI of 5 were transplanted into lethally irradiated recipient mice. (A) The morphology of control and Pxn-KD platelets was examined by transmission electron microscopy, and the areas of granules and cytoplasm in each platelet were independently quantified using ImageJ software for Macintosh. Columns and error bars represent the mean ± s.d. (n = 53–70). (B–C) Washed platelets were lysed to measure the concentrations of platelet factor 4 (PF4) (B) and serotonin (C). Columns and error bars represent the mean ± s.d. (n = 4). Statistical significance was determined using Student’s t test. ***P < 0.001 vs. control. [file 1477-9560-12-1-S3.pdf]

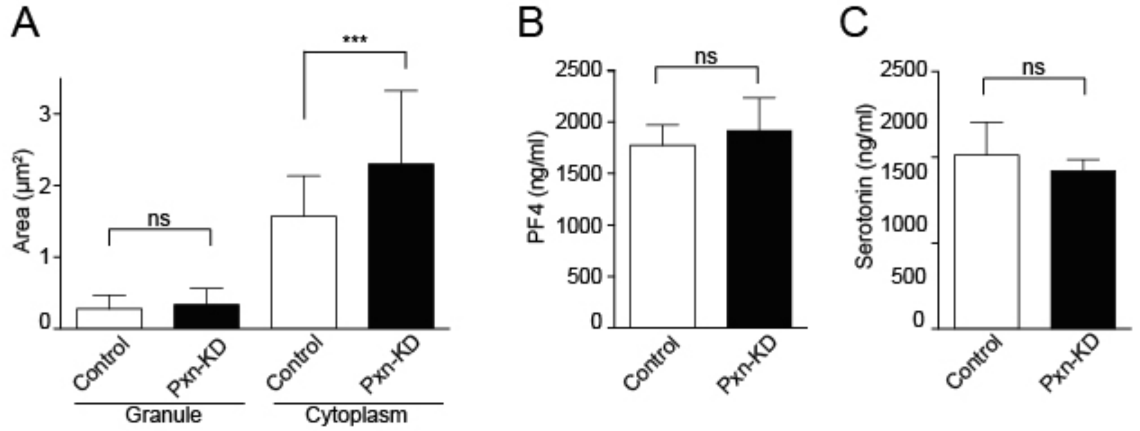

Additional File 3. Pxn-KD does not affect granule contents. Bone marrow cells transduced with LentiLox-sh-control-GPIbα (Control) or LentiLox-sh-paxillin-GPIbα (Pxn-KD) at an MOI of 5 were transplanted into lethally irradiated recipient mice. (A) The morphology of control and Pxn-KD platelets was examined by transmission electron microscopy, and the areas of granules and cytoplasm in each platelet were independently quantified using ImageJ software for Macintosh. Columns and error bars represent the mean ± s.d. (n = 53–70). (B–C) Washed platelets were lysed to measure the concentrations of platelet factor 4 (PF4) (B) and serotonin (C). Columns and error bars represent the mean ± s.d. (n = 4). Statistical significance was determined using Student's t test. ***$P < 0.001$ vs. control.
