# Supplementary material for: Paxillin is an intrinsic negative regulator of platelet activation in mice
Source: Thromb J. 2014 Jan 2;12:1. doi: 10.1186/1477-9560-12-1 (PMC3904695; doi:10.1186/1477-9560-12-1)
Supplement: Additional file 4 — Expression levels of platelet-specific glycoproteins. Description of data: (A) Expression levels of GPIIb/IIIa (integrin αIIbβ3) (left panel), GPIb (middle panel), and GPVI (right panel) in control (dark gray) and paxillin-knockdown platelets (light gray). (B) Columns and error bars represent the mean ± s.d. of the mean fluorescence intensity (MFI) of antibody binding (n = 5). Statistical significance was determined using Student’s t test. *P < 0.05, **P < 0.01, and ***P < 0.001 vs. control. [file 1477-9560-12-1-S4.pdf]

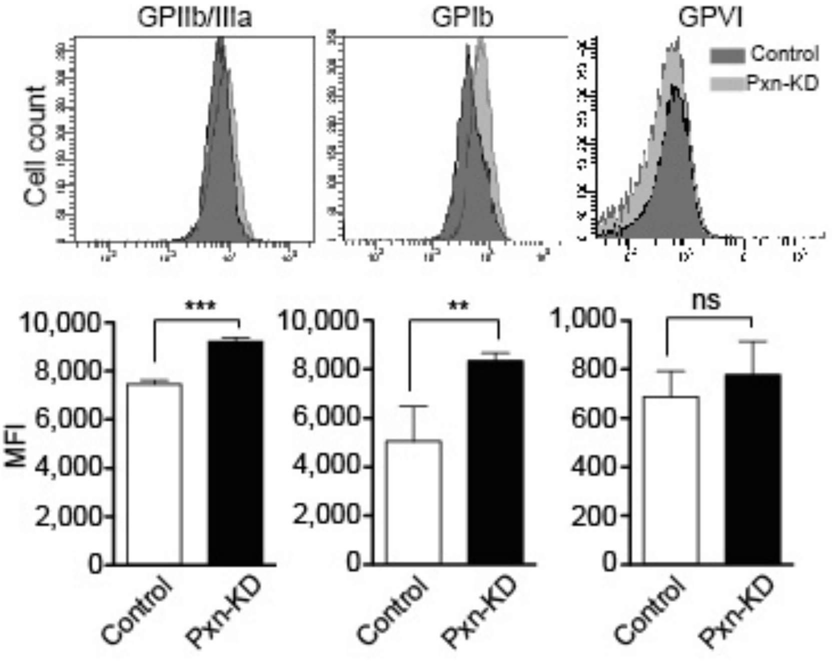

Additional File 4. Expression levels of platelet-specific glycoproteins. (A) Expression levels of GPIIb/IIIa (integrin αIIbβ3) (left panel), GPIb (middle panel), and GPVI (right panel) in control (dark gray) and paxillin-knockdown platelets (light gray). (B) Columns and error bars represent the mean ± s.d. of the mean fluorescence intensity (MFI) of antibody binding ($n = 5$). Statistical significance was determined using Student's t test. $^*P < 0.05$, $^{**}P < 0.01$, and $^{***}P < 0.001$ vs. control.
