# Supplementary material for: Paxillin is an intrinsic negative regulator of platelet activation in mice
Source: Thromb J. 2014 Jan 2;12:1. doi: 10.1186/1477-9560-12-1 (PMC3904695; doi:10.1186/1477-9560-12-1)
Supplement: Additional file 5 — Effects of apyrase and SQ29548 on agonist-induced integrin αIIbβ3 activation and P-selectin expression in control and Pxn-KD platelets. Platelets pretreated without or with 5 U/mL apyrase and 10 μmol/L SQ29548 were stimulated with the indicated agonists. JON/A binding (A) and P-selectin expression (B) on GFP-positive platelets were assessed by flow cytometry. Column and error bars represent the mean ± s.d. of the mean fluorescence intensity (MFI) (n =3–4). Statistical significance was determined using Student’s t test. *P < 0.05, **P <0.01, and ***P < 0.001 vs. control. [file 1477-9560-12-1-S5.pdf]

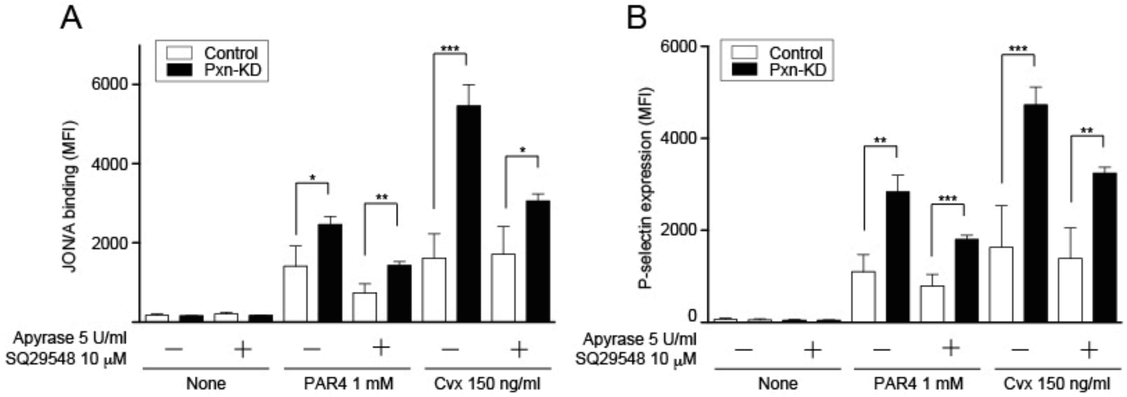

Additional File 5. Effects of apyrase and SQ29548 on agonist-induced integrin αIIbβ3 activation and P-selectin expression in control and Pxn-KD platelets. Platelets pretreated without or with 5 U/mL apyrase and 10 μmol/L SQ29548 were stimulated with the indicated agonists. JON/A binding (A) and P-selectin expression (B) on GFP-positive platelets were assessed by flow cytometry. Columns and error bars represent the mean ± s.d. of the mean fluorescence intensity (MFI) (n = 3–4). Statistical significance was determined using Student's t test. *$P < 0.05$, **$P < 0.01$, and ***$P < 0.001$ vs. control.
