# Supplementary material for: Paxillin is an intrinsic negative regulator of platelet activation in mice
Source: Thromb J. 2014 Jan 2;12:1. doi: 10.1186/1477-9560-12-1 (PMC3904695; doi:10.1186/1477-9560-12-1)
Supplement: Additional file 8 — Thrombus formation in femoral arteries induced by FeCl3. (A) Intravital imaging of thrombus formation 5 mins after FeCl3 treatment in femoral arteries in mice with control or paxillin knock-down platelets (Pxn-KD). The black arrows indicate the direction of blood flow, and triangles show the developed thrombus. Bar, 100 μm. (B) Areas of thrombus within arteries 20 mins after laser irradiation. Columns and error bars represent the mean ± s.e.m. (n = 8 arteries in four mice/group). [file 1477-9560-12-1-S8.pdf]

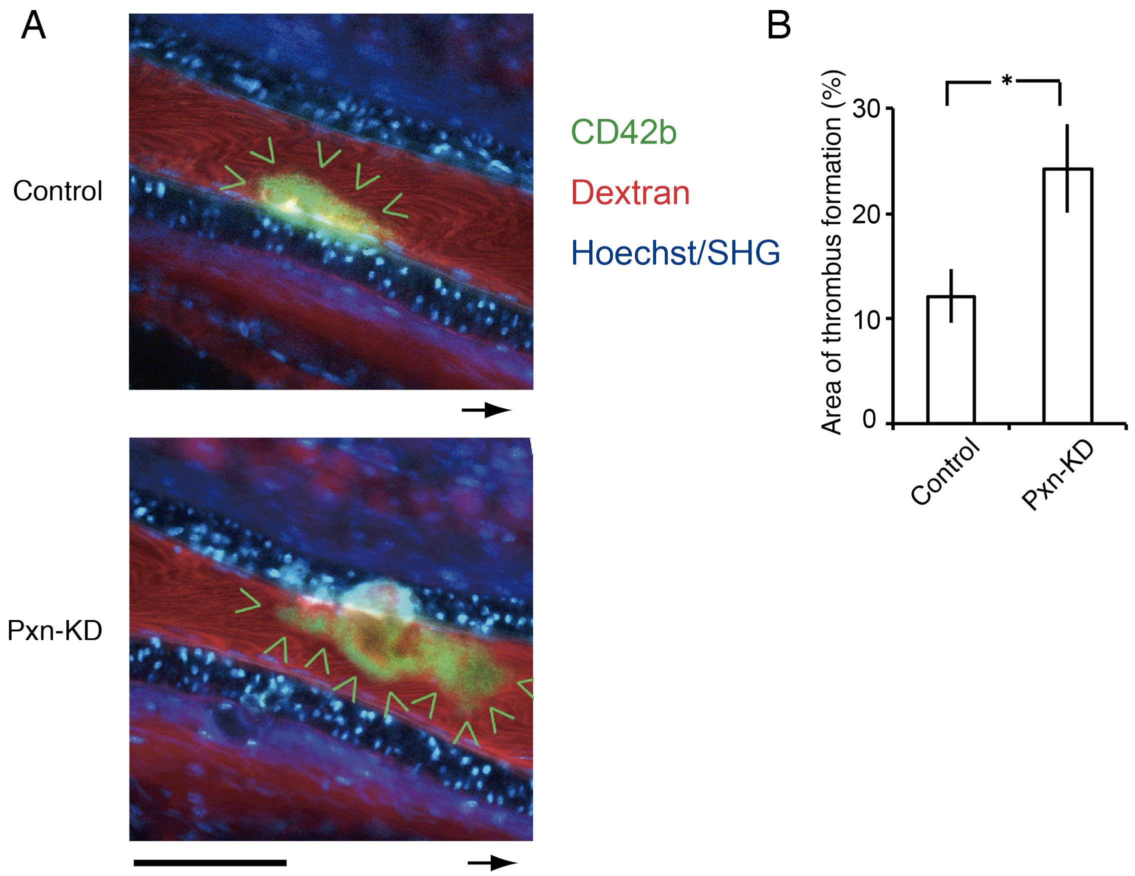

Additional Figure 5. Thrombus formation in femoral arteries induced by $FeCl_3$. (A) Intravital imaging of thrombus formation 5 mins after $FeCl_3$ treatment in femoral arteries in mice with control or paxillin knock-down platelets (Pxn-KD). The black arrows indicate the direction of blood flow, and triangles show the developed thrombus. Bar, 100 μm. (B) Areas of thrombus within arteries 20 mins after laser irradiation. Columns and error bars represent the mean ± s.e.m. (n = 8 arteries in four mice/group).
