# Supplementary material for: Paxillin is an intrinsic negative regulator of platelet activation in mice
Source: Thromb J. 2014 Jan 2;12:1. doi: 10.1186/1477-9560-12-1 (PMC3904695; doi:10.1186/1477-9560-12-1)
Supplement: Additional file 9 — Knock-down of paxillin does not affect talin-dependent activation of integrin αIIbβ3 in CHO cells. (A) Schematic representation of the lentiviral vectors used in this experiment. (B–D) αIIbβ3-CHO cells were transduced with lentiviral vectors expressing a control shRNA sequence and GFP (Control), the paxillin shRNA sequence and GFP (Pxn-KD), a control shRNA sequence and the GFP-Talin FERM domain (Control-FERM), or the paxillin shRNA sequence and the GFP-Talin FERM domain (Pxn-KD-FERM). (B) Lysates obtained from the transduced cells were immunoblotted with anti-GFP polyclonal antibody, anti-paxillin monoclonal antibody, and anti-vinculin monoclonal antibody. (C) PAC-1 binding after transduction in the presence or absence of 1 mmol/L GRGDS was assessed by flow cytometry. Data are representative of four independent experiments. (D) Columns and error bars represent the mean ± s.d. of PAC-1 binding (n = 4). Statistical significance was determined using Student’s t test. [file 1477-9560-12-1-S9.pdf]

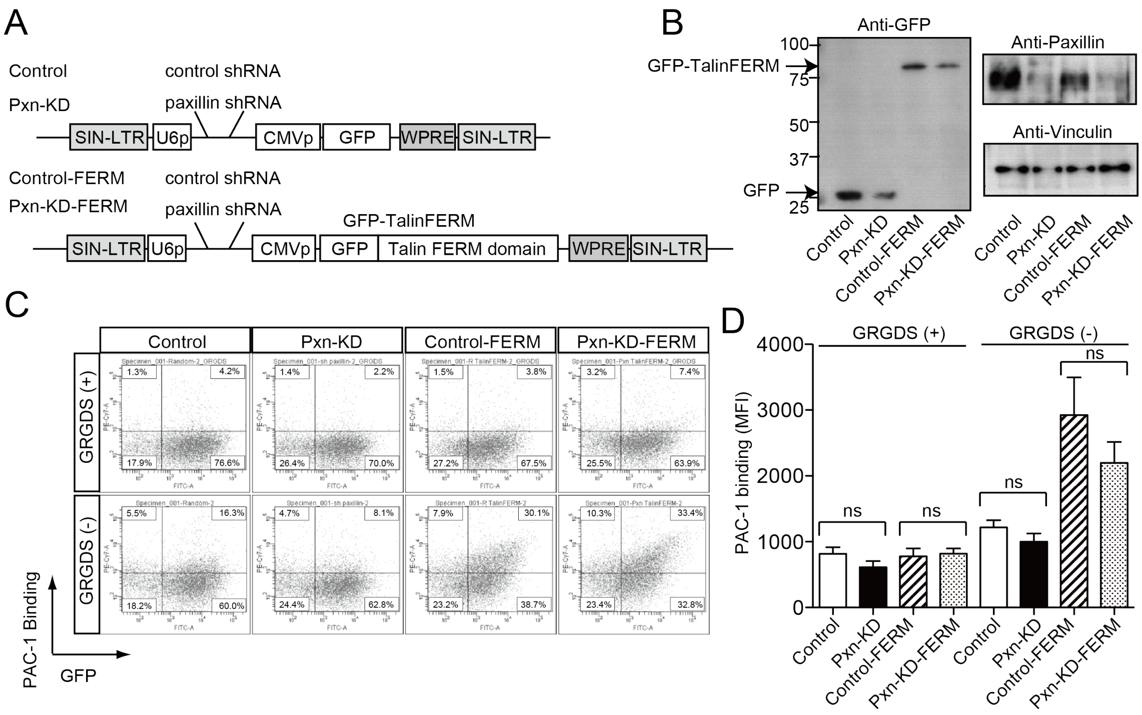

Additional Figure 6. Knock-down of paxillin does not affect talin-dependent activation of integrin αIIbβ3 in CHO cells.

(A) Schematic representation of the lentiviral vectors used in this experiment. (B–D) αIIbβ3-CHO cells were transduced with lentiviral vectors expressing a control shRNA sequence and GFP (Control), the paxillin shRNA sequence and GFP (Pxn-KD), a control shRNA sequence and the GFP-Talin FERM domain (Control-FERM), or the paxillin shRNA sequence and the GFP-Talin FERM domain (Pxn-KD-FERM). (B) Lysates obtained from the transduced cells were immunoblotted with anti-GFP polyclonal antibody, anti-paxillin monoclonal antibody, and anti-vinculin monoclonal antibody. (C) PAC-1 binding after transduction in the presence or absence of 1 mmol/L GRGDS was assessed by flow cytometry. Data are representative of four independent experiments. (D) Columns and error bars represent the mean ± s.d. of PAC-1 binding (n = 4). Statistical significance was determined using Student's t test.
